# Disinfection of Bacteria in Water by Capacitive Deionization

**DOI:** 10.3389/fchem.2020.00774

**Published:** 2020-08-31

**Authors:** Karthik Laxman, Priyanka Sathe, Mohammed Al Abri, Sergey Dobretsov, Joydeep Dutta

**Affiliations:** ^1^Functional Materials Group, Department of Applied Physics, School of Engineering Sciences (SCI), KTH Royal Institute of Technology, Stockholm, Sweden; ^2^Nanotechnology Research Centre, Sultan Qaboos University, Muscat, Oman; ^3^Department of Marine Science and Fisheries, College of Agricultural and Marine Sciences, Sultan Qaboos University, Muscat, Oman; ^4^Department of Petroleum and Chemical Engineering, College of Engineering, Sultan Qaboos University, Muscat, Oman; ^5^Center of Excellence in Marine Biotechnology, Sultan Qaboos University, Muscat, Oman

**Keywords:** capacitive deionization, water treatment, desalination, antibacterial, disinfection

## Abstract

Clean water is one of the primary UN sustainable development goals for 2,030 and sustainable water deionization and disinfection is the backbone of that goal. Capacitive deionization (CDI) is an upcoming technique for water deionization and has shown substantial promise for large scale commercialization. In this study, activated carbon cloth (ACC) electrode based CDI devices are used to study the removal of ionic contaminants in water and the effect of ion concentrations on the electrosorption and disinfection functions of the CDI device for mixed microbial communities in groundwater and a model bacterial strain *Escherichia coli*. Up to 75 % of microbial cells could be removed in a single pass through the CDI unit for both synthetic and groundwater, while maintaining the salt removal activity. Mortality of the microbial cells were also observed during the CDI cell regeneration and correlated with the chloride ion concentrations. The power consumption and salt removal capacity in the presence and absence of salt were mapped and shown to be as low as 0.1 kWh m^−3^ and 9.5 mg g^−1^, respectively. The results indicate that CDI could be a viable option for single step deionization and microbial disinfection of brackish water.

## Introduction

Desalination and disinfection are the two most important aspects related to the production of clean water in several regions of the world. Desalination refers to the reduction of salt content in water, while disinfection relates to neutralization of microbial species in water (World Health Organization, 2011). While desalination is primarily known for production of potable water from seawater, the declining fresh-water quality globally has increased its utilization in a wide array of applications, including that of municipal and groundwater desalination for producing drinking water. Membrane based processes like reverse osmosis (RO), nanofiltration, and ultrafiltration are widely accepted in the drinking water market and it is not uncommon for these systems to de-couple the desalination and disinfection functions (Wang Y. H. et al., 2019). Typically, RO membranes are used to reduce the ionic content of water and work in conjunction with other techniques which reduce the organics and microbial content (disinfection) of the water prior to it being passed through the RO membrane. Managing the biological matter is a pre-requisite to reduce the membrane biofouling propensity of the water and subsequent propagation of water-based infections (Al-Abri et al., 2019; Wang Y. H. et al., 2019).

Technologies for water disinfection have constantly evolved with time, from simple sedimentation processes to chemical treatments and advanced oxidation techniques including chlorination and ozonation (von Gunten, 2003; Richardson and Postigo, 2012; Ding et al., 2019). Among them, chlorination is the most widely used technique providing both primary and residual disinfection, albeit with certain disadvantages (Kimbrough and Suffet, 2002; Hua and Reckhow, 2007; Richardson and Postigo, 2012; Al-Abri et al., 2019; Stefán et al., 2019; Zhong et al., 2019). However it is fast being replaced with new technologies like photocatalysis, UV light treatment, ultrasonication, magnetic enhanced disinfection, and electrochemical methods (Baruah et al., 2012; Bora et al., 2017; Cheema et al., 2018; Cho et al., 2019; Jiang et al., 2019; Wang C. et al., 2019; You et al., 2019). Please note that most of the new methods are good primary disinfectants which kill/disable the microbes instantly upon contact, but not for residual disinfection, which demands persistence of the disinfection process over time.

It is evident that a single step process and its associated technology which can provide both desalination and disinfection would be a step forward in the water purification sector. This is where Capacitive Deionization (CDI) becomes interesting, as it has been demonstrated to desalinate and disinfect water (Laxman et al., 2015a). CDI is an offshoot of the better known electrochemical oxidation, wherein an electric potential is used to produce short lived Reactive Oxygen Species (ROS, namely ·OH, ClO^−^ etc.) in water, which increases the microbial mortality, providing both primary, and residual disinfection mechanisms (Gusmão et al., 2009; Gil et al., 2019). However, unlike the electrochemical technique, it has been argued that since CDI uses a lower DC voltage, limited number of ROS species would be produced (Kim et al., 2019). CDI typically comprises of two high surface area and electrically conductive electrodes, usually different forms of porous activated carbons, like powders, fabric, aerogels etc. separated by a non-conductive spacer (Suss et al., 2015; Ahmed and Tewari, 2018; Oladunni et al., 2018; Choi et al., 2019; Teow and Mohammad, 2019). When a low DC potential (typically <2.0 V_DC_) is applied across the electrodes, non-Faradaic processes occur, leading to the electrosorption of charged species in water (cations and anions) onto the electrode surfaces, resulting in deionization of the input water (Nordstrand et al., 2019; Tang et al., 2019). Following similar principles, the negative charge on the Gram-positive and Gram-negative bacterial cell wall mediates their adsorption onto the positively charged electrode surface through electrosorption processes, thus removing the microbes from the treated water (Laxman et al., 2015a; Wang et al., 2018; Yasin et al., 2018). The inter-electrode electric field magnitude and the total surface area of the electrodes available for electrosorption are important in determining the charged species removal efficacy of CDI devices (Laxman et al., 2015b, 2019). While CDI systems have been well-studied for removing a variety of ionic contaminants from water, the dynamics of microbial removal, especially in the presence of ionic species has not been reported.

In this study, we investigated the potential of an activated carbon cloth (ACC) based capacitive deionization (CDI) cell for the removal of high concentrations (10^7^ CFU mL^−1^) of a Gram-negative coliform bacterium *Escherichia coli* from synthetically prepared water with various salt (NaCl) concentrations. The adsorption dynamics for *E. coli* and the effect of competing ionic species on the bacterial removal and disinfection capacity were investigated by using *E. coli* spiked water. Additionally, removal of Gram-positive and Gram-negative bacteria from inland brackish water collected from a well in Oman was tested. The relative ion adsorption capacities, power consumption and anti-microbial activity of the CDI device were evaluated to assess the viability of using CDI for groundwater deionization and disinfection.

## Materials and Methods

### Chemicals and Materials

Groundwater with pH 8.0 (at 28°C) and a total dissolved solids (TDS) level of 2,500 mg L^−1^ was obtained from Al Musanaah wilayat (North Western Oman, 23.7474°N, 57.6326°E). Analytical grade nitric acid 65% and sodium chloride were purchased from MERCK (Germany) and used as obtained. Double woven activated carbon cloth (ACC) with a thickness of 1.0 mm (Brasquet and Le Cloirec, 1999; Shim et al., 2001) was obtained from Chemviron (Zorflex FM-100). Prior to its use as electrodes in the CDI device, it was cleaned overnight with 2 M nitric acid (heated to 115°C), after which it was thoroughly rinsed with copious amounts of deionized water and dried in a vacuum oven at 150°C.

### Capacitive Deionization (CDI) Cell

The CDI cell comprised of two 25 cm^2^ ACC electrodes separated by a cellulosic spacer. A 50 ml reservoir made up of poly-methyl methacrylate (PMMA) (Dow Corning SYLGARD® 184 Silicone elastomer kit), two graphite rod current collectors and an acrylic plate for support comprised the rest of the CDI cell. Uniformity in potential distribution was maintained by inserting flat graphite sheets between the electrical contacts (acting as current collectors) and the ACC electrodes. The water flow was maintained in a mixed mode (flow-through and flow between), while the applied potential and current data were extracted from the current collectors.

### Deionization Experiments

#### Experiments With *E. coli*

**Figure 1:**
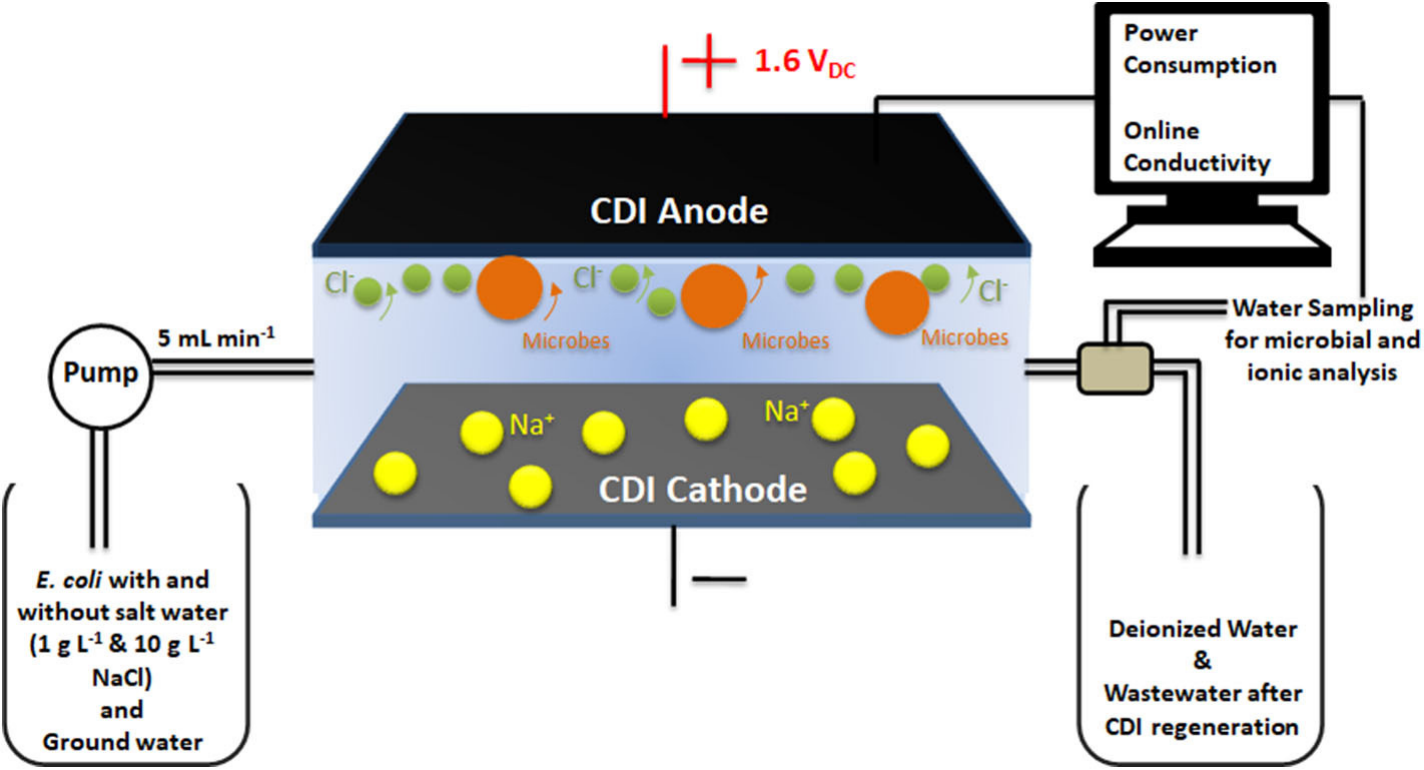
Representation of the experimental set-up comprising of the CDI cell in continuous flow mode and water sampling to measure the microbial and ionic content in water outflow.

The adsorption/desorption experiments were conducted with the Gram-negative bacterium *Escherichia coli* (ATCC 25922) and synthetic (distilled, DI) water with different salt concentrations 0, 1 and 10 g L^−1^ NaCl. Bacteria were cultured in Luria Bertani (LB) broth (Difco, USA) at 37°C for 12 h. Then, the bacterial cells were centrifuged at 5,000 rpm at 25°C for 10 min and re-suspended in feed solution of DI water with different salt concentrations or without salt. The final concentration of bacteria in feed solution was 2.5 × 10^7^ CFU mL^−1^. The experiments were conducted with continuous water flow, where bacterial cells mixed with feed solutions were passed through the cell at a flow rate of 5 mL min^−1^ in order to carry out the disinfection processes under operating conditions found optimal for standard deionization processes. A peristaltic pump (Heidolph pump drive 5201) was used to maintain a constant flow rate of the feed water into the CDI cell. During the deionization/adsorption cycle, a DC potential of 1.6 V_DC_ was applied across the CDI electrodes and water coming out from CDI unit was sampled at various stages of the deionization cycle. Samples were used for counting of live bacteria (Figure 1). The charging current dynamics and potential voltage across the cell were monitored using Gwinstek GDM-396 online multimeters. Change in conductivity was recorded using eDAQ ET 908 online conductivity probe with a cell volume of 93 μL.

#### Groundwater Experiments

For groundwater deionization and disinfection, brackish water [TDS 2.5 g L^−1^ (Cond. = 1.6 g L^−1^); pH 8.0] with a microbial content of 3 × 10^4^ CFU mL^−1^ was collected from a well in Oman's Al Musanaah Wilayat (north western Oman, GPS coordinates 23.48.21 N, 57.34.10 E). Besides, *E. coli*, this water was found to contain other Gram-negative and positive bacteria, such as *Bacillus* spp., *Shigella* spp., *Vibrio* spp., *Klugiella* spp., *Coccus* spp., and *Polynucleobacter* spp. For groundwater, the deionization cycle was continued until the point of minimum conductivity was reached, after which the electrode was regenerated. The cation adsorption efficiencies were observed by collecting water samples (10 mL) at the point of minimum conductivity and measured using an ICP-OES after a 10-fold dilution. Bacterial electrosorption efficiencies were studied by collecting 10 mL of samples during deionization. The number of live bacteria in the sample were determined by the number of colony forming units (CFU, see below) in the desalinated water samples. Electrode regeneration was carried out by electrically shorting the electrodes while continuing to flow the feed water through the CDI cell. The regeneration cycle was continued until original feed water conductivity was reached.

#### Determination of the Number of Live Bacterial Cells

The number of live bacteria in samples were determined by counting the number of colony forming units (CFU) and live and dead staining (Al-Hinai et al., 2017; Sathe et al., 2017). To determine CFU, serial 10-fold dilutions of collected groundwater or synthetic water samples were made with sterile distilled water. 0.1 mL of the sample from each serial dilution was plated onto each Petri dish containing sterile nutrient agar (Difco, USA). The plates were incubated at 37°C for up to 48 h to permit microbial growth. Colonies were counted manually after 48 h. The concentration of live bacterial cells in CFU mL^−1^ was calculated using the following formula.

$$\begin{array}{l} \text{CFU m}{\text{L}}^{-1}=\frac{Number\;of\;colonies\;x\;Dilution\;factor}{Plated\;volume\;in\;mL} \end{array}$$

The number of live and dead cells in collected synthetic water samples with different salinity and groundwater samples were additionally counted by an epifluorescent microscope (Zeiss, Germany) at 1,000 × magnification (Al-Hinai et al., 2017). One hundred microliters of collected samples were immediately stained with the LIVE/DEAD BacLight^TM^ kit (Molecular Probes, USA), which is a mixture of SYTO 9 and propidium iodide dyes in DMSO. SYTO 9 preferentially stains cells in green color with intact membranes while PI stains cells in red color with damaged membranes, enabling the quantification of live, and dead cells in the water samples. The stained samples were applied on a microscope slide and representative pictures of randomly selected fields of view (area = 0.001 mm^2^) were made by a camera (Muthukrishnan et al., 2017).

#### Characterization of CDI Electrodes and Ionic Composition of Water

Active surface area of ACC was calculated by a nuclear magnetic resonance (NMR) relaxation technique in Xigo Nanotools, using water as the solvent. The changes in the T2 relaxation time was measured and analyzed by Acorn area software (Xigo Nanotools, NJ, USA) to calculate the active surface area of the electrode (using the specific surface relaxivity on activated carbon). Zeta potential measurements of the flat electrodes were carried out in SurPass Electrokinetic analyzer (Anton Paar, Austria). The electrolyte concentration was maintained at 0.001 M KCl. Electrode size was kept constant with an L × W of 20 mm × 10 mm and the gap between the two electrodes was maintained at ~100 μm for all measurements. The electrolyte flow was ramped from 0 to 400 mbar with a ramping duration of 20 s, while the rinsing period was maintained at 5 min for all measurements. Each zeta potential was determined by averaging four measurements. Electrode surface was studied by imaging in JEOL JSM-7200 field emission scanning electron microscope (FESEM, JEOL, Japan) working at 20 kV. Specific salt adsorption capacity was calculated using the following formula:

$$\begin{array}{l} \Gamma =\frac{\left({\text{C}}_{\text{o}}-\text{C}\right)\text{ V}}{\text{M}} \end{array}$$

Where, ‘Γ’ is the electrosorption capacity in mg g^−1^, “C_o_” is the initial salt concentration in mg L^−1^, “C” is the minimum concentration in mg L^−1^, “V” is the volume of NaCl solution passing through the cell during deionization in “L,” and “M” is the total mass of the electrodes in grams.

Cation concentrations were determined by Varian 710-ES inductively coupled plasma optical emission spectrometer (ICP-OES, Varian, CA, USA). The power consumed was calculated by integrating the charging current to obtain the total charge. The charge was then multiplied by the applied potential (1.6 V_DC_) to obtain the work done in joules, which was subsequently converted to kilo-watt hour (kWh) units per 1,000 liters (1 m^−3^) of water treated.

## Results

### ACC Electrode Characterization

NMR surface area measurements indicated that the activated carbon cloth (ACC) electrode used for the experiments has an active surface area of 980 m^2^ g^−1^. Based on reports available from Zorflex, the FM-100 ACC is predominantly microporous with a mean pore diameter of ~0.7 nm. The total pore volume for ACC is 1.417 cm^3^ g^−1^, with micropores accounting for 0.56 cm^3^ g^−1^, macropores for 0.85 cm^3^ g^−1^ and mesopores for <0.001 cm^3^ g^−1^. While this porosity is well-suited for ionic adsorption, its efficacy for bacterial cell removal within a mixed water matrix which comprises of multiple ionic and organic/microbial species needs to be evaluated. Secondly, the overpotential of ACC for water electrolysis and gas evolution (which typically takes place at 1.23 V vs. SHE) needs to be determined to ensure that the applied DC potential is limited to values at which ACC surface oxidation is not enhanced.

**Figure 2:**
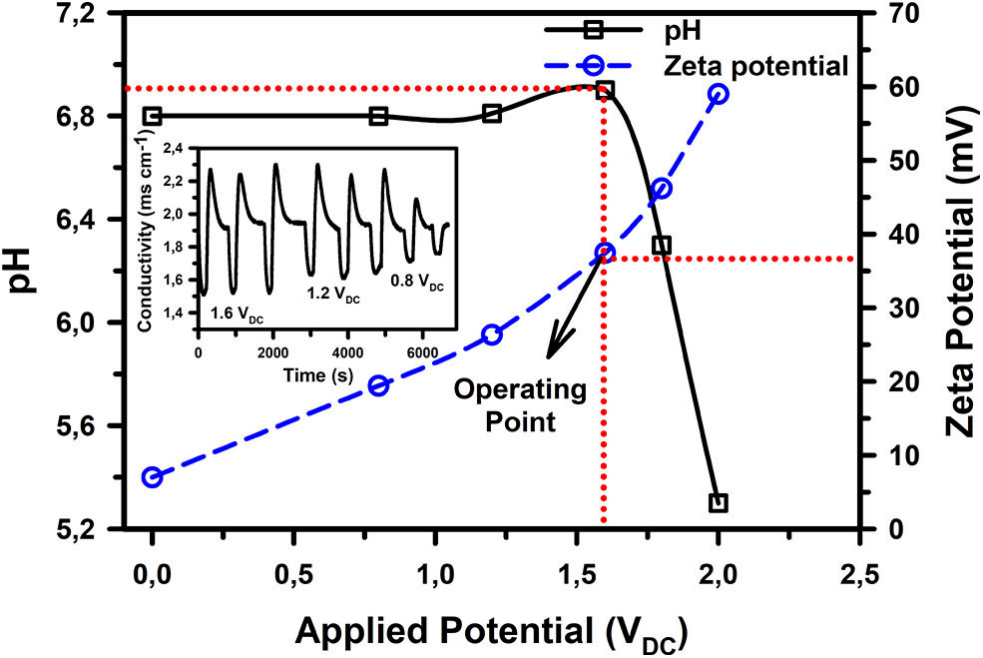
Change in ACC surface zeta potential and pH of the exit solution with applied potential at 25°C. Inset shows the continuously varying conductivity curve of a 1 g L^−1^ NaCl solution at potentials of 1.6, 1.2, and 0.8 V_DC_.

As can be observed from Figure 2, the overpotential for ACC, which is a function of the device construction (cell interfaces and power transfer efficiency) and material properties extends the noticeable electrolysis potential for water to ~1.6 V_DC_ (at a peak current density of 1.25 mA cm^−2^), above which faradaic reactions become significant enough to bring about a noticeable change in the water pH. Nonetheless, the surface zeta potential and ion adsorption capacity of ACC were observed to be directly proportional to the applied DC voltage (Figure 2 and insert). This provides a plausible justification to operate the CDI unit at the upper potential limit of 1.6 V_DC_, wherein we have previously observed good ion removal capacity (Laxman et al., 2015a, 2019).

### *E. coli* Removal From Deionized Water

**Figure 3:**
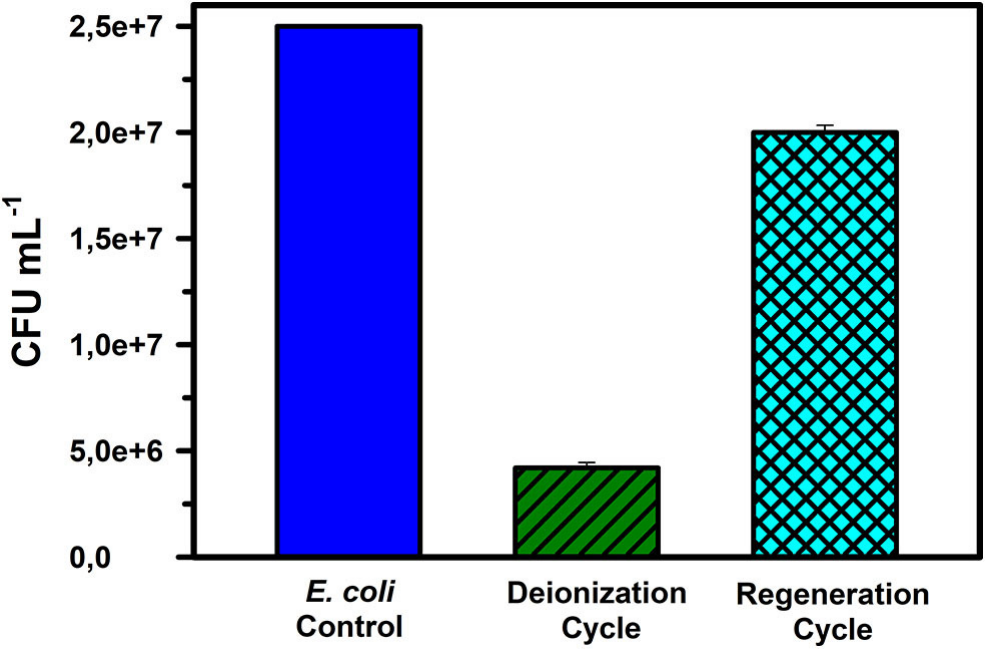
The number of live *E. coli* cells expressed as CFU mL^−1^ counts for samples in the reservoir (control), during deionization, and during regeneration cycles in DI water. The data are the means of 3 replicates + standard errors.

At the applied potential of 1.6 V_DC_, the *E. coli* removal capacity was observed to be ~85 % from an initial concentration of 2.5 × 10^7^ CFU mL^−1^ in DI water (Figure 3). Staining images (as discussed later) indicate that both the absolute density of bacterial cells and the viable cells were reduced in the desalinated water stream, while simultaneously, the number of dead bacterial cells increased in the regenerated water samples. During electrode regeneration conducted under short-circuit conditions, the concentration of live bacteria was close to 80% of the control (inlet microbial concentration) value. Since this was a continuous flow mode experiment with *E. coli* culture, the results indicate that only 15% of the microbes escaped the treatment during deionization and even during regeneration, the CFU mL^−1^ of water exiting the CDI device was 20% lower than the CFU mL^−1^ of incoming water (control) (Table 1).

**Table 1:** Ionic and microbial removal efficiencies of CDI during deionization and regeneration for well-water and synthetic water with 1 g L^−1^ and 10 g L^−1^ NaCl concentrations.

**Parameter**	**DI Water + *E. coli* culture**	**1 g L^−1^ NaCl + *E. coli* culture**	**10 g L^−1^ NaCl + *E. coli* culture**	**Groundwater**
Na^+^ removal (0.36 g L^−1^)	–	–	–	11%
Ca^2+^ removal (0.13 g L^−1^)	–	–	–	63%
K^+^ removal (0.009 g L^−1^)	–	–	–	46%
Al^3+^ removal (0.012 g L^−1^)	–	–	–	84%
Reduction in CFU mL^−1^ (Deionization)	85%	64%	62%	67%
Reduction in CFU mL^−1^(Regeneration)	20%	60%	75%	33%
Spec. salt ads. Capacity	–	6.1 mg g^−1^	9.5 mg g^−1^	5.9 mg g^−1^
Power consumption	0.1 kWh m^−3^	0.4 kWh m^−3^	0.73 kWh m^−3^	0.48 kWh m^−3^

*The numbers in brackets in column 1 represent the initial concentration of the well water ions in g L^−1^*.

### Effect of Salt on *E. coli* Removal

**Figure 4:**
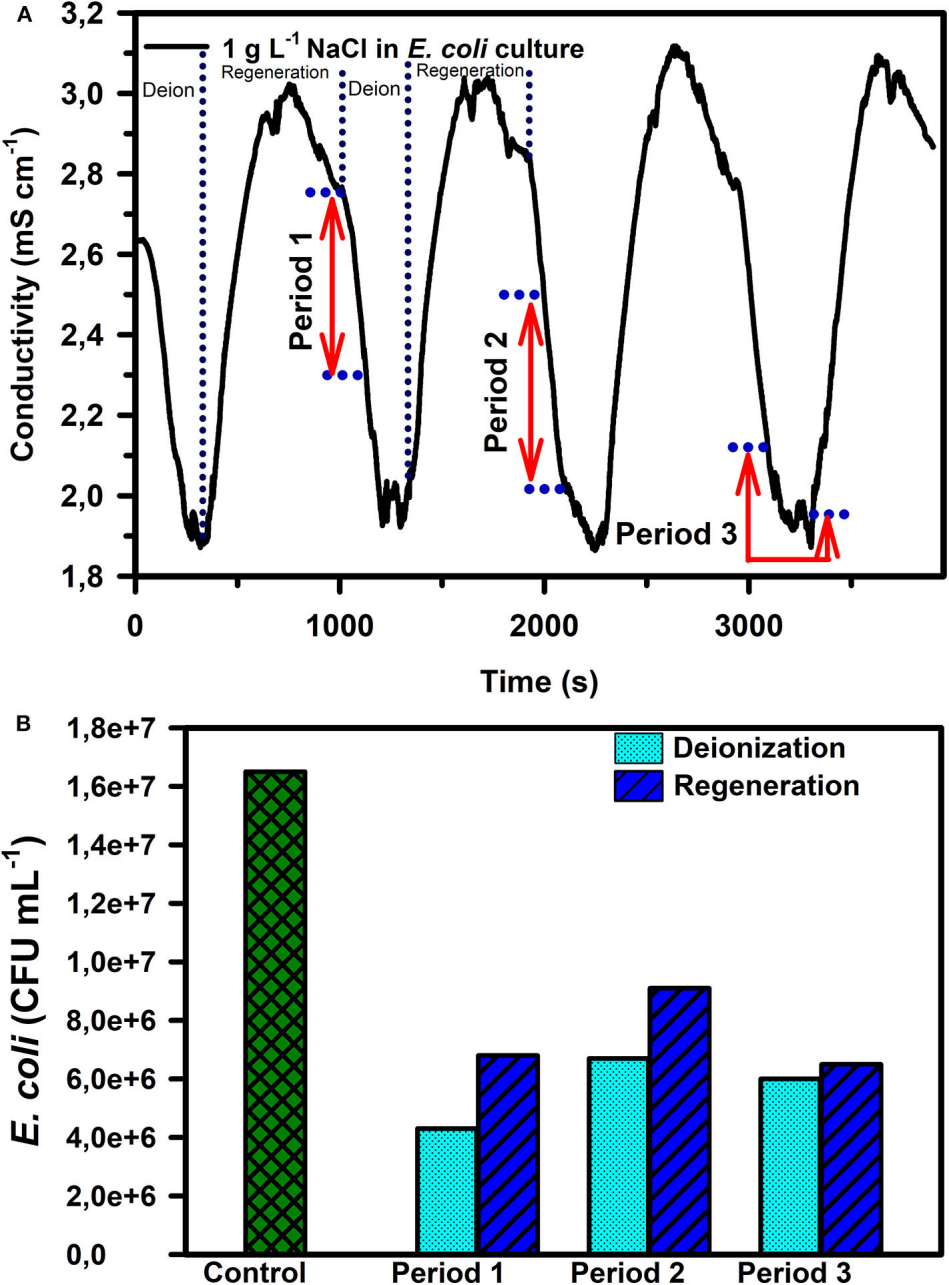
**(A)** Conductivity curve during deionization and regeneration cycles for a 1 g L^−1^ NaCl in *E. coli* culture and **(B)** *E. coli* CFU mL^−1^ for different sample collection periods marked in **(A)**. The reported measurements in **(B)** are an average of 3 repetitions with the following standard error percentages from the mean (± 5.5, ± 2.0, & ± 4.0% for deionization periods 1, 2, and 3 respectively and ± 1.9, ± 5.0, & ± 7.0% during regeneration for periods 1, 2, and 3, respectively).

*E. coli* removal capacity of CDI in the presence of two different concentrations of NaCl (1 g L^−1^ and 10 g L^−1^) was also characterized. In general, the conductivity curve for both the NaCl concentrations showed an initial fast decay indicating a high rate of charged species electrosorption/neutralization. However, the slope of the conductivity curve keeps decreasing with time until a quasi-steady state is reached which indicates the rate of electrosorption and release of charged species is identical. After this period, the conductivity curve starts rising again as a result of the reduced kinetics of electrosorption as the electrode is moving toward saturation. For these experiments, the CDI devices were switched to regeneration mode once quasi-steady state was reached. During regeneration, the desorption of ions from electrodes leads to a rise in the electrical conductivity of water as observed in Figure 4A. The water samples collected during three different deionization periods (Figure 4A) were analyzed and it was observed that the number of live bacteria was lowest in period 1 (Figure 4B), compared to periods 2 and 3.

**Figure 5:**
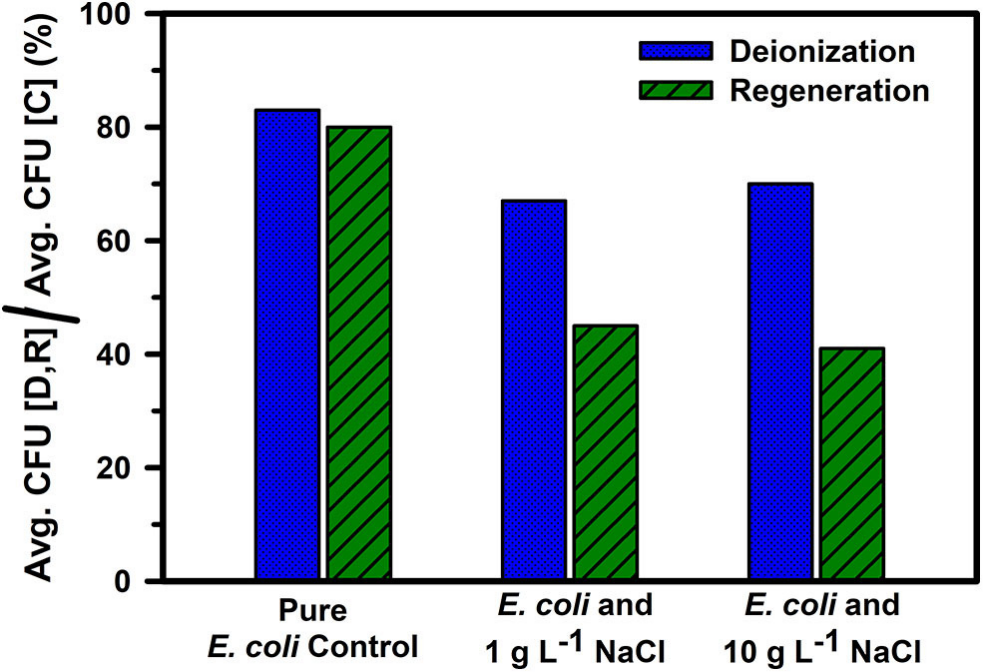
Comparison of ratio of viable *E. coli* cells after the deionization and regeneration for *E. coli* in DI water (control) and with 1 g L^−1^ and 10 g L^−1^ NaCl concentrations with the control sample. “D,” “R,” and “C” stand for deionization, regeneration, and control, respectively. Averages were calculated over 4 deionization and regeneration cycles.

**Figure 6:**
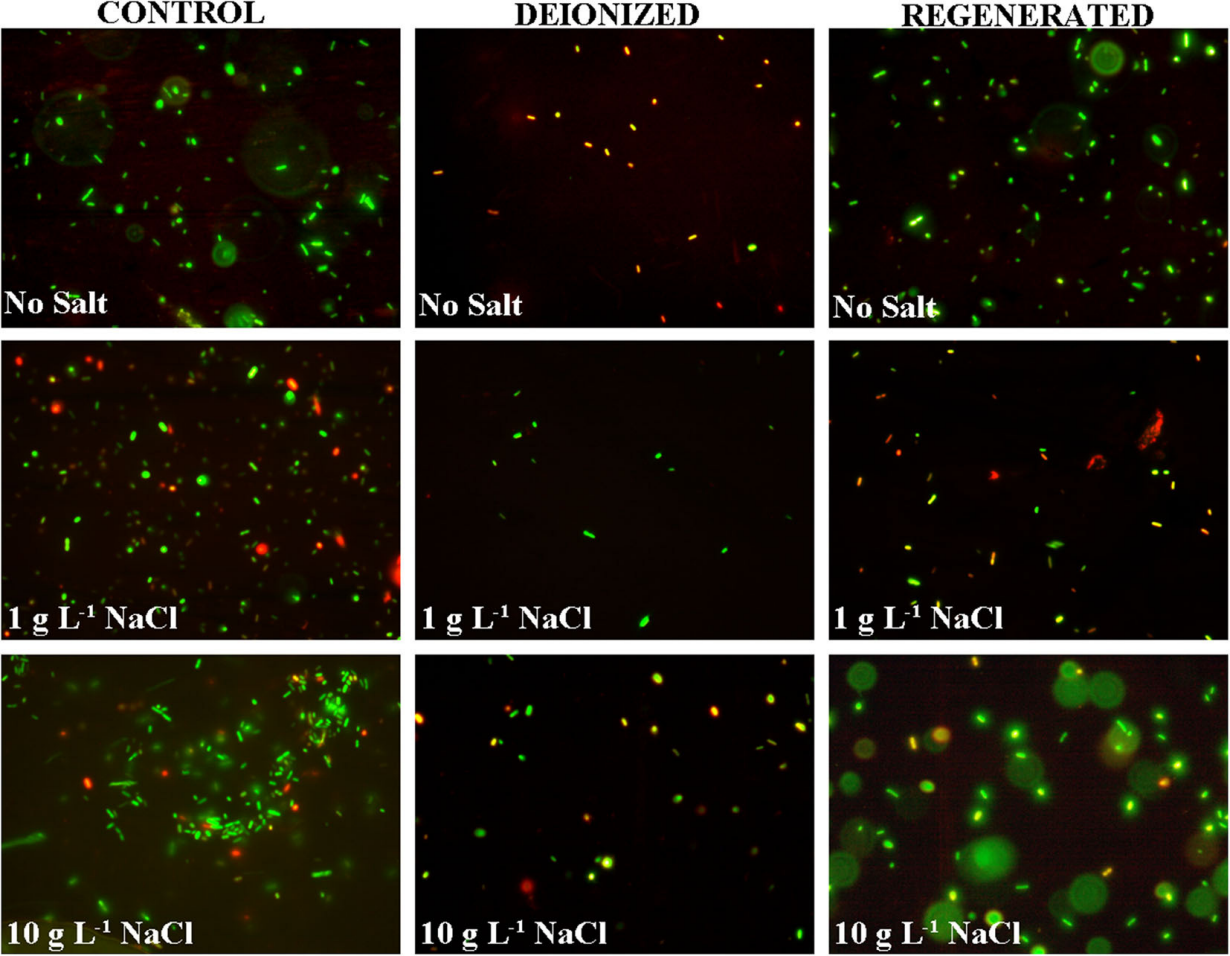
Live and dead stained *E. coli* cells in the control, deionized (after CDI), and regenerated water samples for DI water, and DI water with 1 g L^−1^ and 10 g L^−1^ NaCl.

The addition of salt reduced the microbial neutralization capacity of the electrodes to ~64% at 1 g L^−1^ salinity and 62% at 10 g L^−1^ salinity (Figure 5 and Table 1). This is also supported by Figure 6, where it is clear that the presence of salt in the *E. coli* water samples increases the number of live bacteria during the deionization. Thus, the electrosorption and chemical neutralization capacity of the deionization cycle on *E. coli* are affected depending on the absence and presence of salt. However, it can be observed that the absolute amount of salt or the ionic strength of the solution seemed to have little effect on the electrosorption and neutralization process (Lytle et al., 1999).

However, during CDI cell regeneration, there were differences observed in the survival and mortality of *E. coli* cells in water with different salinity levels (Figures 5, 6). While 20% of incoming bacterial cells were neutralized during regeneration phase of CDI electrodes for *E. coli* in DI water, the relative percentages of neutralized cells increased to 60% with 1 g L^−1^ of NaCl in DI water and 75% for 10 g L^−1^ NaCl in DI water, respectively (Table 1 and Figures 5, 6). Some mortality of *E. coli* was also observed in the control treatment with 1 g L^−1^ and 10 g L^−1^ NaCl, due to the fact that it is a non-halophilic bacterium (Kunin et al., 1992).

### Groundwater Decontamination

Analysis of the groundwater samples indicated that it mainly contained a mixture of monovalent, divalent and trivalent ions along with microbial cells (Table 1). The ions were electrosorbed at the electrodes to different extents during the deionization period. Ions with a higher oxidation number were adsorbed more effectively as they present a more energetically favorable option for screening the electrode surface charge (Hou and Huang, 2013; Li et al., 2016). However, when the charge on the ions was similar, the absolute concentrations took precedence, wherein ions with higher concentration like Na^+^ are electrosorbed to a larger extent as compared to ions with lower concentrations like K^+^ (Table 1) (Nordstrand and Dutta, 2020). In addition to *E. coli*, the groundwater contains other bacterial species, such as *Bacillus* spp., *Shigella* spp., *Vibrio* spp., *Klugiella* spp., *Coccus* spp., and *Polynucleobacter* spp. Among these, *E. coli, Shigella* spp., and *Salmonella* spp. are the main pathogens that are known to cause serious diseases outbreaks (Pandey et al., 2014). During deionization, the number of viable microbial cells in groundwater was reduced by ~67% from 3 × 10^4^ CFU mL^−1^ to 1 × 10^4^ CFU mL^−1^. During electrode regeneration, the number of viable microbes was found to increase 2-fold (to 2 × 10^4^ CFU mL^−1^), which is essentially a 33% reduction in the CFU counts (Table 1). The SSA of ACC is found to be 5.9 mg g^−1^ with a power consumption of 0.48 kWh m^−3^ of water treated (Table 1). It is noteworthy to observe that the power consumption and SSA of groundwater and low salinity synthetic water (1 g L^−1^ NaCl) contaminated with *E. coli* were comparable (Table 1). As expected, the SSA and power consumption for the high salinity synthetic water sample (10 g L^−1^ NaCl) was much larger at 9.5 mg g^−1^ and 0.73 kWh m^−3^, respectively (Table 1).

## Discussion

Based on results obtained in Figure 3, we hypothesize that the strong electric field and acidic water pH in the vicinity of the positive electrode (where microbes are adsorbed) may be responsible for the bacterial cell mortality during deionization and regeneration. Previously it has been shown that electrical field causes irreversible permeabilization of bacterial cells (Feng et al., 2004; del Pozo et al., 2009). Additionally, even though no change in water pH was observed during the experiment, faradaic reactions are known to occur at the electrodes, which could lead to the formation of hydrogen peroxide (H_2_O_2_) and hydronium ions (H_3_O^+^) at the CDI cathode and anode, respectively (He et al., 2016). These chemical oxidants deactivate *E. coli* cells during the deionization phase and are released into the water stream during regeneration of the electrodes. Removal of bacterial cells by electrically generated reagent species have been reported earlier (Durán Moreno et al., 2004; Wang et al., 2014). The mechanisms stated above are a function of the operating conditions like applied potential (1.6 V_DC_) and fluid flow rate (at 5 ml min^−1^ or specific flow rate of 0.13 mL min^−1^ cm^−2^ of electrode surface area). Together, they resulted in 85% mortality of the *E. coli* cells. The mortality % is of course subject to change depending on modifications in the above-mentioned parameters.

However, most natural water samples, including groundwater, comprise of a mixed matrix of ionic, organic, and microbial contaminants (Chilton, 1996). The results in the manuscript clearly indicate that the presence of ionic matter can alter the bacterial electrosorption and neutralization processes in a CDI device. Primarily, this is due to the better electrosorption kinetics of the smaller and more mobile ionic species, which can compete with the microbial cells for binding sites on the electrode surface. As a result, the electric field strength is lowered quickly, essentially reducing its effect on the microbes. However, simultaneously, the higher concentration of ionic species at the electrode surfaces may very well create an environment for dehydration of the microbials cells present at the electrode surfaces (Li et al., 2002). Thus, ionic species can have both a positive and negative effect on microbial neutralization. Figure 4A shows that the initial fast conductivity transition period (period 1) has the highest mass transfer rate of ions toward the electrode surface (Demirer et al., 2013), which reduces with time as the electrode approaches saturation (Nordstrand and Dutta, 2019, 2020). The lower quantity of live bacterial cells in period 1 (Figure 4B) is plausible as the electric field strength between the CDI device electrodes is the strongest during period 1. This period 1 action on *E. coli* cells also indicates that irrespective of the size and charge type (electronic vs. chemical), the action on the ions and microbial cells, be it electrosorption or neutralization through electro-chemical action, is proportional to the electric field strength and possibly to the surface charge. It should be noted that while *E. coli* cells are larger and have a slower mass transfer rate compared to the ions, the outer layer of lipopolysaccharides on the Gram-negative cell imparts a strong negative zeta potential ranging from −23 mV to −50 mV at pH ~7 (Lytle et al., 1999; Martins et al., 2013), which can significantly assist in their migration toward the positive electrode surface. Nonetheless, the net electrosorption/neutralization capacity of CDI is reduced in the presence of salt, which we attribute to the preferential electrosorption of the ions at the electrode surface due to their smaller size. The same result is also reflected visually through fluorescence microscopy images as observed in Figure 6.

Despite lower net *E. coli* neutralization in the presence of salt, there could be multiple mechanisms effecting this observed mortality. The first being electrosorption, wherein the negatively charged bacterial cells will be in the same vicinity of the electrosorbed chloride ions (Cl^−^) (Marugán et al., 2010) and the higher concentrations of Cl^−^ ions may lead to oxidative stress on the cell walls resulting in cell death. Secondly, the electric field may affect electrosorbed bacterial cells (Feng et al., 2004; del Pozo et al., 2009). Thirdly, since the reduction potential for chloride is 1.36 V (vs. SHE), it is possible that some of the Cl^−^ ions get reduced to Cl_2_(g), in turn leading to the generation of hypochlorous acid (HClO), which increases the mortality of bacterial cells in the vicinity of the anode (Zhang et al., 2018). Typically, the formation of HClO has been observed to be proportional to the amount of electrosorbed Cl^−^ ions (Wouters et al., 2013). It should be noted that when the CDI device is switched from deionization to regeneration (at the quasi steady state point), the absolute quantity of electrosorbed Cl^−^ ions for the 1 g L^−1^ and 10 g L^−1^ NaCl samples are comparable. Hence the potential HClO concentrations at the electrode surface are also comparable, which partially justifies the similarity in microbial cell neutralization capacity for the two salinities (Figures 5, 6). In addition, and as mentioned previously, some oxygen based oxidative species (like H_2_O_2_ and H_3_O^+^) may also contribute to be microbial mortality. Based on similar reasoning, the higher mortality of *E. coli* during CDI regeneration in saline water (compared to DI water in Table 1) can be attributed to the release of the chloride and chloride based radical species into the water stream (Zhang et al., 2018), which effectively doubles the chloride concentration in the water stream creating an environment where bacterial cells can be effectively neutralized during regeneration. Hence in summary, a reduction in *E. coli* cell mortality during the deionization phase was only observed between deionized water and 1 g L^−1^ NaCl water (Figures 4, 5). A further 10-fold increase in salt concentration (10 g L^−1^) resulted in mere 15% increase in bacterial cell mortality. On a similar note but contrarily, a significant increase in *E. coli* cell mortality during the regeneration phase was only observed between deionized water and 1 g L^−1^ NaCl water; while a further increase in salinity did not contribute much to the enhancement in mortality percentages.

**Figure 7:**
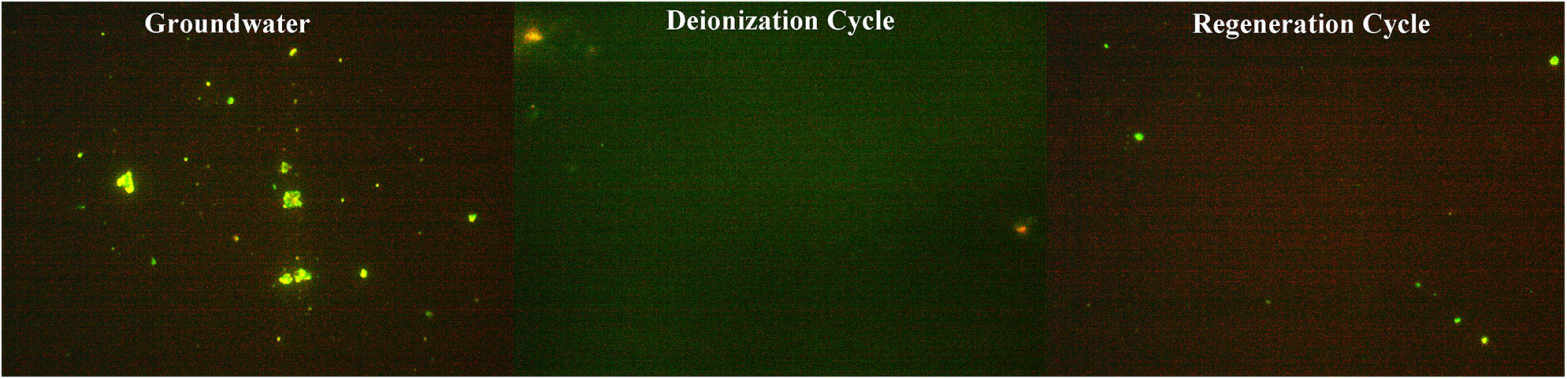
Microbial cells stained with life and dead BacLight^TM^ stain in original, deionized, and regeneration groundwater samples. Salinity of groundwater was ~1.5 g L^−1^ and comprises of multiple ions (Table 1).

For real groundwater samples, the co-occurrence of multiple ions and microbial species, along with the organic contaminants pose different conditions and outcomes for a CDI cell operation (Laxman et al., 2015a). Live and dead staining indicated that the proportion of microbes neutralized during deionization (Figure 7) was similar to the saline water samples prepared in the laboratory (Figure 6), which is feasible, since the salinity percentages were similar. However, during regeneration of groundwater the microbial cell mortality is reduced compared to that of the synthetic samples, which although not certain, could be attributed to the multiple bacterial species in the groundwater which can respond differently to the CDI effect. In addition, the organic load in the water can also modulate the radical formation capacity and in turn hinder the cell neutralization during regeneration. Nonetheless, the results clearly indicate that a CDI device effectively eliminates multiple species of Gram-positive and negative bacteria from the groundwater. However, the extent to which microbes can be removed show dependence on the surface charge, size and sensitivity of the microbial surface charge to electrolyte concentration and pH (Lytle et al., 1999). This would be interesting to study in further details in the future.

In addition, while not much discussion has been given on the electrode performance in the various experiments, it should be noted that the specific salt adsorption capacity (SSA) and power consumption of the CDI device did not vary significantly for the groundwater and *E. coli* spiked NaCl water samples of similar TDS levels (Table 1). SSA and power requirements primarily increases with high salt concentration which allows the electric field to be better neutralized, leading to thinner but denser electrical double layers as compared to the lower salt containing water samples (Suss et al., 2015). Overall there were no surprising results in the electrode performance criteria with the *E. coli* spiked DI water samples showing the lowest power requirements (0.1 kWh m^−3^), followed by 1 g L^−1^ and 10 g L^−1^ *E. coli* spiked NaCl samples.

## Conclusions

In conclusion, activated carbon cloth (ACC) was used as an electrode material in capacitive deionization (CDI) process and the technique was found to be effective for the simultaneous deionization and elimination of microbes from water. This process can be applied in a multi-ion water matrix and is feasible for both pathogenic and biofouling bacteria. The bacterial neutralization during the deionization and regeneration processes was observed to be a function of the presence or absence of salt, but not so much on the absolute salt concentrations. During deionization, bacterial neutralization is proposed to occur through multiple mechanisms due to electric field, osmotic effects and chemical action, while during regeneration, we hypothesize that the close proximity of the bacterial cells to the chloride ions and formation of oxidative oxygen and chloride species in water dominated the neutralization process. Thus, CDI using ACC electrodes is found to be potentially viable for the purification and disinfection of groundwater with reasonably high microbial concentrations. Since CDI does not use synthetic biocides but eliminates bacteria from the water primarily through electrosorption and other naturally occurring radical species in water, this method has a minimal impact on the environment and can be recommended as a green alternative for water treatment for deionization and microbial disinfection.

## Data Availability Statement

All datasets presented in this study are included in the article/supplementary material.

## Author Contributions

KL and PS conducted the experiments and wrote the manuscript. MA, SD, and JD planned the experiments and edited the manuscript. All authors contributed to the article and approved the submitted version.

## Conflict of Interest

The authors declare that the research was conducted in the absence of any commercial or financial relationships that could be construed as a potential conflict of interest.
